# Structural, Functional and Antioxidant Properties of Soy Protein Concentrates Enriched with Black Mulberry Pomace

**DOI:** 10.17113/ftb.64.03.26.9373

**Published:** 2026-09-15

**Authors:** Nevena Barać, Biljana Rabrenović, Ivana Sredović Ignjatović, Steva Lević, Vladimir Pavlović, Slađana Žilić, Danka Milovanović, Miroljub Barać

**Affiliations:** 1 Faculty of Agriculture, University of Belgrade, Nemanjina 6, 11080 Belgrade, Serbia; 2 Maize Research Institute, Slobodana Bajića 1, Zemun polje, 11185 Belgrade, Serbia

**Keywords:** soy protein concentrate, black mulberry pomace, polyphenol-protein interactions, antioxidant properties, techno-functional performance, sustainable co-ingredients

## Abstract

**Research background:**

The incorporation of fruit processing residues into protein-rich ingredients is attracting attention as a sustainable strategy to enhance the nutritional and functional value of plant-based formulations. However, the effect of adding black mulberry pomace on protein matrix structure, functionality and digestibility remains insufficiently explored. This study investigates the potential of black mulberry pomace as a co-ingredient to modify and improve the characteristics of soy protein concentrate derived from moderately toasted soy flour.

**Experimental approach:**

Soy protein concentrates were obtained by washing soy flour with ethanol after blending it with increasing mass fractions of black mulberry pomace. The chemical composition, *in vitro* digestibility, hydration and emulsifying properties, and antioxidant potential of the prepared samples were examined using standard radical scavenging and metal-chelating assays. Structural characteristics were analysed using infrared spectroscopy and electron microscopy. Relationships among compositional, functional, and antioxidant parameters were explored using multivariate statistical analyses.

**Results and conclusions:**

The addition of small amounts of black mulberry pomace improved hydration and emulsifying properties, as well as antioxidant potential through the incorporation of phenolic compounds. These enhancements were attributed to interactions between phenolic compounds and protein structures, confirmed by spectroscopic and microscopic evidence. However, higher pomace mass fractions negatively affected protein digestibility, likely due to matrix densification and aggregation. Correlation and multivariate analyses confirmed close associations between antioxidant activity, protein solubility, and techno-functional performance. These findings suggest that low to moderate pomace mass fractions are sufficient to induce positive changes without compromising digestibility.

**Novelty and scientific contribution:**

This work provides new insights into the dual role of black mulberry pomace as a functional and antioxidant-enhancing agent in plant protein systems. It demonstrates a sustainable approach to developing value-added protein ingredients by integrating underutilised agri-food residues. The comprehensive chemical, functional, and structural analyses presented in this study contribute to a better understanding of how co-ingredient interactions can shape the bio-functional performance of soy-based matrices.

## INTRODUCTION

The growing demand for sustainable, health-promoting food ingredients has increased interest in plant-based proteins as alternatives to animal-derived products. Soy protein is particularly valued for its favourable amino acid composition, high digestibility, and versatile functionality in food systems. Among soy-derived ingredients, soy protein concentrates (SPCs) are widely used for their high protein content and favourable techno-functional properties including water- and oil-holding capacity, emulsifying capacity, and emulsion stability (*1*).

Meanwhile, the agri-food industry generates large amounts of plant-based by-products that are rich in fibre, organic acids, vitamins, minerals and bioactive compounds but often remain underutilised. Recent studies (*2*-*5*) report that pomace from apple, grape, citrus and berries can enhance nutritional value and modulate technological and sensory properties when incorporated into cereal, bakery, dairy, or extruded products. In addition to its fibre fraction, pomace contains polyphenols, including flavonoids and anthocyanins, which exhibit antioxidant, anti-inflammatory, and other bioactivities that can be harnessed to design clean-label, health-oriented foods (*6*, *7*).

Black mulberry (*Morus nigra* L.) pomace, a major residue from juice and wine production, contains abundant polyphenols, flavonoids, and dietary fibre, and demonstrates promising antioxidant potential (*8*). Its valorisation aligns with circular economy principles and offers opportunities to develop novel functional ingredients. However, its potential has not yet been fully explored.

A promising strategy for the next generation of SPCs is the integration of fruit-derived materials during SPC manufacture to co-extract and/or retain bioactive compounds while tailoring functionality. The conventional extraction with alcohol efficiently removes soluble carbohydrates from soy but may also remove endogenous phytochemicals (*1*). Introducing a polyphenol-rich fruit fraction during extraction could offset such losses by supplying additional phenolics and fibre and by enabling *in situ* formation of polyphenol-protein associations. Depending on the phenolic class and processing conditions, these covalent and non-covalent interactions can alter protein conformation, surface hydrophobicity and charge with resulting effects on solubility, gelation, emulsifying activity, and even protein digestibility (*9*, *10*).

In this context, co-processing soy flour with black mulberry pomace during SPC manufacture is expected to yield products with enhanced phenolic content, antioxidant capacity, and potentially improved functional properties, while contributing to sustainable by-product utilisation. The methodology used in this study is particularly relevant because aqueous-ethanol extraction is already a well-established industrial process for SPC production. Integrating fruit pomace at this stage requires no major process redesign yet allows direct incorporation of valuable bioactive compounds into the protein matrix. This approach also provides a unique model system for studying the effects of polyphenol-protein interactions formed under realistic industrial processing conditions.

Therefore, the aim of this study is to investigate the chemical composition and techno-functional, antioxidant, and microstructural properties of SPCs obtained by ethanol extraction of soy flour co-processed with different mass fractions (0–10 %) of black mulberry pomace. Protein and sugar mass fractions, total phenolic content (TPC), total flavonoid content (TFC), colour parameters, antioxidant capacity (DPPH, ABTS), *in vitro* protein digestibility, and functional properties (water- and oil-holding capacity, emulsifying capacity and emulsion stability) were determined. Fourier transform infrared (FTIR) spectroscopy and scanning electron microscopy (SEM) were employed to elucidate structural and surface modifications. This integrated approach is expected to provide new insights into the functionalisation of SPCs through co-processing with polyphenol- and fibre-rich fruit by-products, thereby supporting the development of multifunctional, clean-label plant protein ingredients.

## MATERIALS AND METHODS

### Materials

Moderately toasted defatted soy flour (Bp10L; Bankom doo, Belgrade, Serbia) was used to prepare soy protein concentrates (SPCs). Fresh black mulberry (*Morus nigra* L.) pomace, a by-product obtained after cold-pressed juice production, was collected and stored at −18 °C until further use. Its average chemical composition on a dry matter basis was as follows: total sugars 17.8 %, crude fibre 57.0 %, crude fat 9.5 %, protein 8.7 %, total phenolic content (expressed as gallic acid equivalents (GAE); Sigma-Aldrich, Merck, St Louis, MO, USA) 6.06 mg/g, total flavonoid content (expressed as quercetin equivalents (QE); Sigma-Aldrich, Merck) 2.61 mg/g, and anthocyanin content (expressed as cyanidin-3-glucoside (Cy-3-G) equivalents) 150 μg/100 g (unpublished data).

### Preparation of soy protein concentrates

Defatted soy flour was blended with mulberry pomace at mass fractions of 0, 1, 2, 5 and 10 %. The mixtures were extracted with *φ*(aqueous ethanol)=65 % for 60 min at 40 °C using a magnetic stirrer to ensure continuous agitation. After extraction, the suspensions were centrifuged at 2500×*g* for 10 min (DM0412; DLAB, Beijing, PR China). The resulting pellets were rinsed with 50 mL of the same ethanol solution and centrifuged again under identical conditions. The supernatants were discarded, and the pellets, representing the soy protein concentrates, were dried in a laboratory oven at 50 °C for 6 h. The dried material was then ground into a fine powder using a laboratory mill (A11 Basic; IKA, Staufen, Germany). All samples were prepared in triplicate using the same batches of soy flour and mulberry pomace.

### Chemical composition analysis

The chemical composition of SPCs was determined using standard analytical procedures. Total nitrogen (TN) was analysed using the Kjeldahl method and expressed as total protein per dry matter (*11*). The method is based on acid digestion of the sample, followed by distillation and titration of released ammonia to quantify nitrogen content. Dry matter was determined by oven-drying at 105 °C to constant mass (*12*), while fat content was measured by Soxhlet extraction using *n*-hexane (*13*). Crude fibre content was determined according to the standard AOAC procedure (*14*), based on sequential acid and alkaline digestion of the sample residue.

Water-soluble and acid-hydrolysable sugars were quantified using the phenol-sulphuric acid method (*15*). Briefly, 1 g of SPC sample was extracted with 10 mL of deionised water (Milli-Q, Merck Millipore, Darmstadt, Germany) for 1 h and centrifuged at 2500×*g* (DM0412; DLAB) for 10 min. The supernatant was used to determine water-soluble sugars. The pellet was rinsed with 5 mL of deionised water, centrifuged under the same conditions, and hydrolysed with 10 mL of 0.5 mol/L H_2_SO_4_ in a boiling water bath for 2 h. After cooling, the mixture was centrifuged again, and the supernatant was used to determine acid-hydrolysable sugars. Absorbance was measured at 490 nm using a UV-Vis spectrophotometer (UV-1800; Shimadzu, Kyoto, Japan). Sugar content on dry mass basis was calculated from a glucose calibration curve and expressed as g/100 g.

Water-soluble protein content on dry mass basis was determined using the Bradford method (*16*), with bovine serum albumin (BSA; Sigma-Aldrich, Merck) as the standard. Results were expressed as g/100 g.

### Determination of total phenolic and flavonoid content

Total phenolic content (TPC) of the SPCs was determined using the Folin–Ciocalteu method, as described by Kostić *et al*. (*17*), with slight modifications. Briefly, 0.1 g of powdered SPC was extracted in 10 mL of deionised water using a vortex mixer for 1 h at room temperature. The extracts were centrifuged at 2400×*g* for 10 min (DMO412; DLAB). A volume of 182 μL of undiluted extract was mixed with 909 μL of Folin (Sigma-Aldrich, Merck) working solution (1:9 dilution). After 5 min, 909 μL of 7.5 % sodium carbonate solution were added. The mixtures were kept in the dark for 90 min, after which absorbance was measured at 765 nm using a UV-Vis spectrophotometer (UV-1800; Shimadzu, Kyoto, Japan). TPC was expressed as milligrams of GAE per gram of dry matter, based on a gallic acid calibration curve prepared in the same manner.

Total flavonoid content (TFC) was determined according to the method of Žilić *et al*. (*18*), with slight modifications. A mass of 100 mg of SPC sample was extracted with 10 mL of *φ*(aqueous acetone)=70 % for 30 min using a mechanical shaker, followed by centrifugation at 2500×*g* for 10 min (DMO412; DLAB). The absorbance of the supernatant was measured at 360 nm using a UV-Vis spectrophotometer (UV-1800; Shimadzu). TFC was expressed as milligrams of QE equivalents per gram of dry matter based on a quercetin calibration curve.

### Determination of total anthocyanin content

Total anthocyanin content (TAC) was determined according to the pH differential method as suggested by Giusti and Wrolstad (*19*). Briefly, 2 g of SPC sample was extracted with 20 mL of 95 % ethanol containing 1 % (by volume) HCl. The mixture was stirred on a mechanical shaker for 1 h at room temperature and then centrifuged at 2500×*g* (DMO412; DLAB) for 10 min. The absorbance of the clear supernatant was measured at 520 and 700 nm (UV-1800; Shimadzu) in two buffer systems (potassium chloride buffer, pH=1.0, and sodium acetate buffer, pH=4.5) using a quartz cuvette with a 1 cm path length. TAC was calculated using the following equation:


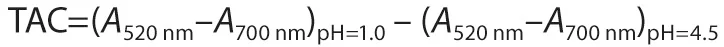
 /1/

The results were expressed as mg of cyanidin-3-glucoside equivalents per 100 g of dry sample using a molar absorption coefficient (*ε*=26.900 L/(mol·cm)) and a molecular mass of 449.2 g/mol.

### Determination of antioxidant properties

The antioxidant properties of SPCs enriched with mulberry pomace were assessed by evaluating Fe(II) chelation, ABTS and DPPH radical scavenging activities. Fe(II) chelating ability was determined according to the method of Meira *et al*. (*20*). Briefly, 100 mg of powdered SPC were extracted with 10 mL of deionised water using a vortex mixer for 30 min, followed by centrifugation at 2500×*g* for 15 min (DM0412; DLAB). A volume of 1 mL of the clear extract was mixed with 3.7 mL of deionised water, 0.1 mL of 2 mmol/L FeSO_4_, and 0.2 mL of 5 mmol/L ferrozine (Sigma-Aldrich, Merck). After 10 min, the absorbance was measured at 562 nm. A control sample was prepared by replacing the extract with deionised water. The chelating ability (%) was calculated as follows:


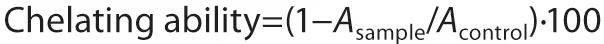
 /2/

ABTS radical scavenging activity was assessed using the method of Arnao *et al*. (*21*). A stock solution of ABTS (Sigma-Aldrich, Merck) was prepared by mixing 7 mmol/L ABTS with 2.45 mmol/L dipotassium peroxydisulfate and allowing it to stand in the dark for 16 h. The working solution was obtained by diluting the stock solution with methanol to an absorbance of 0.70–0.80 at 734 nm. Then, 1 mL of the ABTS working solution was mixed with 50 μL of SPC extract, vortexed, and incubated in the dark for 7 min. A control was prepared using 50 μL of deionised water instead of the extract. Absorbance was measured at 734 nm. The ABTS scavenging activity (%) was calculated as follows:


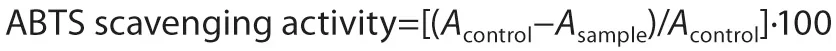
 /3/

A calibration curve was prepared using Trolox (Sigma-Aldrich, Merck) standards (10-100 μg/mL), and the results were expressed as μmol of Trolox equivalents per g of dry matter.

DPPH radical scavenging activity was evaluated according to Kostić *et al*. (*17*). A volume of 105 μL of water extract was mixed with 840 μL of freshly prepared DPPH (Merck, Darmstadt, Germany) working solution. The mixture was incubated in the dark for 30 min, and the absorbance was measured at 515 nm (UV-1800; Shimadzu). The DPPH scavenging capacity was expressed as μmol of Trolox equivalents per g of dry sample.

### Colour measurement

Colour parameters (CIELab) were measured using a Chroma Meter CR-400 (Konica Minolta, Tokyo, Japan), calibrated with a white standard (CM-A70). Ground samples were placed in an optical glass cell and measured using SpectraMagic NX software (*22*). Chroma (C*), hue angle (*h°*) and total colour difference (Δ*E*) were calculated as follows:


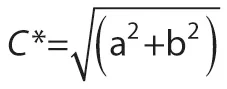
 /4/


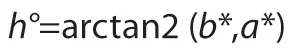
 /5/


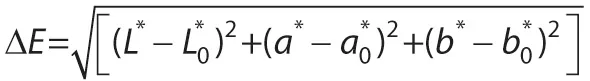
 /6/

where L* is lightness, a* is the red/green coordinate (positive values indicate redness, negative values greenness), b* is the yellow/blue coordinate (positive values indicate yellowness, negative values blueness), C* is chroma (colour saturation), *h*° is hue angle (tone of the colour), Δ*E* is total colour difference between treated sample and the control (0 % black mulberry pomace), and *L*_0_*, *a*_0_* and *b*_0_* are the colour parameters of the control sample. Results were reported as mean values from three independent measurements.

### Determination of techno-functional properties

Emulsifying properties of SPC were assayed using the turbidimetric method as described by Barać *et al*. (*23*). Briefly, pure sunflower oil (15 mL) and 45 mL of a 1 % aqueous suspension of SPC were homogenised in a mechanical homogeniser (MSE Homogeniser, MSE, Crawley, UK) for 1 min at the highest setting (7500×*g*). A volume of 50 μL of emulsions was pipetted from the bottom of the container at 0 and 10 min after homogenisation and diluted with 10 mL of 0.1 % SDS (Sigma-Aldrich, Merck, St Louis) solution. The absorbance of these diluted emulsions was measured at 500 nm. Emulsifying activity index (EAI) and emulsifying stability index (ESI) were calculated using absorbance values at 0 (*A*_0_) and 10 min (*A*_10_) after emulsion formation. Emulsifying properties were expressed as EAI (m^2^/g) and ESI (min) according to the following equations:


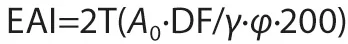
 /7/

where T is 2.303, *A*_0_ is the absorbance measured immediately after emulsion formation, DF is the dilution factor 200, *γ* is the protein mass concentration (g/mL) of the aqueous phase before emulsion formation, and *φ* is the oil volume fraction (calculated by drying the emulsion).


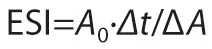
 /8/

where Δ*t* is 10 min and Δ*A* is *A*_0_−*A*_10_.

Water-holding capacity (WHC) and oil-holding capacity (OHC) were assayed as suggested by Gouw *et al*. (*24*). In a Falcon tube, 0.5 g of SPC was mixed with 10 mL of pure sunflower oil, left overnight at room temperature, centrifuged (DMO412; DLAB) at 1500×*g* for 5 min, and the excess oil was carefully decanted. The sample with the adsorbed oil was reweighed, and OHC was expressed as g oil per g dry matter.

In a Falcon tube, 1 g of SPC was mixed with 50 mL of deionised water for 5 min, centrifuged at 4000×*g* (DMO412: DLAB) for 10 min, the excess water was carefully decanted, and the sample was reweighed. The WHC was expressed as g water per g dry matter.

### In vitro multistep enzymatic digestion

Digestibility of SPCs was assessed using a multistep *in vitro* digestion protocol simulating oral, gastric, duodenal and colonic phases based on Papillo *et al*. (*25*) with modifications from Hamzalıoğlu and Gökmen (*26*). Simulated salivary (SSF), gastric (SGF), and duodenal fluids (SDF) were prepared accordingly. A mass of 5 g of sample was sequentially treated with SSF, SGF with pepsin (pH=2.0, 2 h, 37 °C; Sigma-Aldrich, Merck, St Louis), SDF with pancreatin and bile salts (pH=7.5, 2 h, 37 °C), followed by protease (1 h) and Viscozyme L (16 h; Novozymes, Bagsværd, Denmark) incubations at 37 °C with shaking. The digested residue was filtered, air-dried (2 h), and oven-dried at 105 °C (4 h) to constant mass. Digestibility was expressed as the percentage reduction of dry mass.

### Fourier transform infrared spectroscopy

Fourier transform infrared (FTIR) spectra of SFCs were recorded at room temperature using an FTIR spectrophotometer IRAffinity-1 (Shimadzu). Before analysis, the samples were mixed with potassium bromide and compressed into pellets using a hydraulic press. All spectra were recorded in the spectral range 4000–600 cm^-1^, at a resolution of 4 cm^-1^. Each sample was scanned 100 times, and a background was recorded before analysing each sample with a pure KBr pellet. Peak positions (*ν*_mAX_) for O-H, C-H, amide I, amide II, amide III, and C-O stretching bands were identified *via* peak-picking. Shift (Δ*ν*) was calculated relative to the control (0 % pomace). The intensity ratio *I*_1650_/*I*_1540_ was calculated as the ratio of absorbances at amide I and amide II, as a proxy for secondary structural assessment, following standard FTIR methodologies (*27*).

### Scanning electron microscopy

The surface morphology of the samples was examined using a scanning electron microscope (JSM-6390LV; JEOL Ltd., Tokyo, Japan). Before analysis, the samples were mounted on aluminium stubs using double-sided carbon tape and sputter-coated with a thin layer of gold to improve conductivity. Micrographs were captured at various magnifications (100×, 300×, 500× and 1200×) under high vacuum mode and an accelerating voltage of 15 kV.

### Statistical analysis

All experiments were conducted in triplicate, and results are expressed as mean±standard deviation (S.D.). One-way analysis of variance (ANOVA) followed by Tukey’s honestly significant difference (HSD) *post hoc* test was performed to assess significant differences (p<0.05) among sample groups using IBM SPSS Statistics v. 26.0 (*28*). Multivariate analyses, including principal component analysis (PCA), Pearson’s correlation analysis, and hierarchical cluster analysis (HCA), were carried out using Python programming language v. (*29*) with relevant packages: pandas for data handling, scikit-learn for PCA and clustering, and seaborn for data visualisation.

## RESULTS AND DISCUSSION

### Chemical composition of soy protein concentrates

The chemical composition of soy protein concentrates (SPCs) obtained by ethanol extraction from defatted soybean flour blended with different mass fractions of black mulberry pomace is shown in Table 1. Enrichment with pomace significantly affected protein, carbohydrate, fibre and oil fractions (p<0.05).

**Table 1:** Chemical composition of soy protein concentrates with different mass fractions of black mulberry pomace

*w*(pomace)/%	*w*(TP)/(g/100 g)	*w*(SP)/(g/100 g)	w(WSS)/(g/100 g)	*w*(AHS)/(g/100 g)	*w*(fibre)/(g/100 g)	*w*(oil)/(g/100 g)	*w*(moisture)/%
0	(71.9±0.3)^c^	(5.71±0.02)^c^	(0.33±0.01)^d^	(6.4±0.0)^e^	(4.8±0.1)^e^	(0.75±0.02)^c^	(11.2±0.2)^a^
1	(74.4±0.5)^b^	(9.9±0.2)^a^	(0.43±0.06)^c^	(12.7±0.1)^d^	(5.4±0.2)^d^	(0.82±0.04)^b^	(10.3±0.2)^b^
2	(76.1±0.4)^a^	(9.6±0.3)^a^	(0.6±0.0)^b^	(14.43±0.03)^c^	(6.1±0.2)^c^	(0.87±0.07)^b^	(11.1±0.1)^a^
5	(72.6±0.6)^c^	(9.53±0.08)^a^	(0.65±0.01)^a^	(17.2±0.2)^b^	(8.0±0.3)^b^	(1.2±0.1)^a^	(10.6±0.1)^b^
10	(72.4±0.1)^c^	(8.66±0.03)^b^	(0.7±0.0)^a^	(18.9±0.9)^a^	(11.3±0.2)^a^	(1.30±0.05)^a^	(10.5±0.3)^b^

TP=total proteins, SP=soluble proteins, WSS=water-soluble sugar, AHS=acid-hyrolysable sugar, all expressed on dry mass basis, and fibre. Values are mean±S.D. Different letters in superscript in the same column indicate statistically significant differences (p<0.05)

The control sample (0 % pomace) contained 71.92 g/100 g of protein, which is consistent with previously reported values (≥70 %) for ethanol-washed SPCs (*30*). The highest protein mass fraction was observed at 2 % pomace, significantly (p<0.05) exceeding all other samples. At higher pomace mass fractions (5 and 10 %), total protein mass fraction decreased, approaching values similar to those of the control. This biphasic trend suggests that moderate pomace addition may favour protein retention, while larger mass fractions exert a dilution effect associated with increased fibre incorporation. The soluble protein fraction followed a different trend: all enriched samples showed significantly (p<0.05) higher values (8.66–9.89 g/100 g) than the control (5.71 g/100 g). This enhancement is most likely due to the release of low-molecular-mass proteins and their potential interactions with polyphenols, which may unfold protein structures and improve solubility (*9*).

Carbohydrates also reflected the influence of pomace. Water-soluble sugars doubled from 0.33 g/100 g in the control to 0.67 g/100 g at 10 % pomace, while acid-hydrolysable sugars tripled (6.40 to 18.86 g/100 g). These data indicate that the pomace introduced both easily extractable and more resistant polysaccharide fractions. Fibre increased markedly and proportionally to pomace mass fraction, from 4.82 to 11.34 g/100 g, consistent with the fibrous composition of mulberry by-products. Beyond nutritional benefits, this enrichment is expected to modulate techno-functional behaviour such as hydration and oil-binding, and to contribute to physiological effects related to digestive regulation and glycaemic control (*31*, *32*).

Oil content increased more moderately, from 0.75 g/100 g (control) to 1.30 g/100 g (10 % pomace). The additional lipids originated from the seed fraction of mulberry pomace, reported to contain 27.5–33 % crude oil rich in linoleic acid (~73.7 %), with smaller amounts of palmitic, oleic and stearic acids, along with α-tocopherol and phytosterols (*33*). These compounds are known for their antioxidant and cholesterol-lowering properties (*34*).

These results show that adding mulberry pomace to SPC increased the content of soluble protein, fibre, sugars and minor lipophilic compounds. The addition of around 2 % appears optimal for maximising protein yield, whereas higher mass fractions progressively enrich the carbohydrate and fibre fractions, setting the basis for changes observed in techno-functional, antioxidant, and digestibility properties.

### Techno-functional properties

The effects of black mulberry pomace incorporation on the techno-functional properties of SPCs are shown in Fig. 1. Considering the compositional differences described above, these properties were evaluated in terms of water-holding capacity (WHC), oil-holding capacity (OHC), emulsifying activity index (EAI) and emulsion stability index (ESI).

**Fig. 1:**
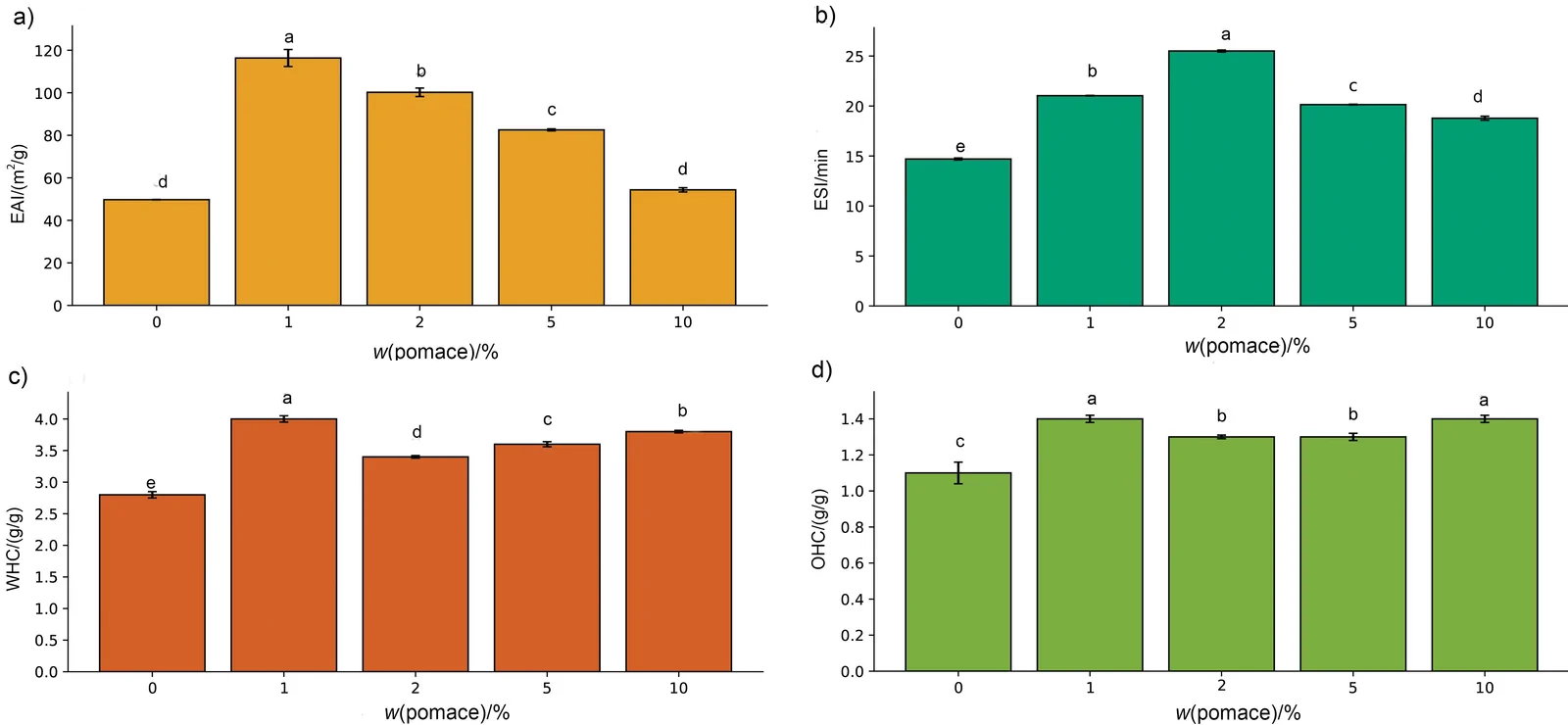
Techno-functional properties of soy protein concentrates enriched with 0, 1, 2, 5 and 10 % black mulberry pomace: a) emulsifying activity index (EAI), b) emulsion stability index (ESI), c) water-holding capacity (WHC), and d) oil-holding capacity (OHC). Values are expressed as mean±S.D. Different letters indicate significant differences (p<0.05). All values are reported on a dry matter basis

WHC exhibited a non-linear trend with the highest value at pomace mass fractions of 1 % (Fig. 1c), followed by a decrease at 2 %. At higher pomace mass fractions (5 and 10 %), WHC values increased again, reaching (3.82±0.02) g/g at 10 %. This pattern indicates that a small to moderate fibre fraction most effectively enhances hydration capacity, likely through the formation of a denser protein-fibre network and the presence of more hydrophilic binding sites. At 10 %, WHC remained higher than that of the control, reflecting the strong contribution of total fibre content and its swelling ability. Comparable trends have been reported for mulberry pomace-fortified yoghurt, where WHC improved relative to the control. Du *et al*. (*5*) showed that the addition of mulberry pomace at 1–3 % increased water-holding capacity by approx. 10–20 %. A comparable pattern was noted for OHC, which was highest at 1 and 10 % inclusion ((1.42±0.02) g/g), while intermediate values (2 and 5 %) remained constant at 1.3 g/g (Fig. 1d). This suggests that both moderate and higher inclusions provide sufficient hydrophobic sites (fibre porosity and exposure of hydrophobic protein regions *via* polyphenol interactions) for oil retention, while intermediate amounts appear saturated. Such functional enhancements are consistent with the results of Elleuch *et al*. (*35*). These authors highlight that plant-derived fibre can promote oil binding through combined structural (porosity, surface area) and surface property (hydrophobic interactions) effects, a mechanism that can also operate in composite systems containing proteins.

The EAI increased significantly with pomace incorporation (p<0.05), reaching a maximum at 1 % inclusion ((116.4±4.0) m^2^/g; Fig. 1a). Samples with 2 and 5 % pomace also showed significantly improved emulsifying activity compared to the control. However, at 10 % pomace, the EAI decreased slightly, although it remained higher than the control. A similar trend was observed for ESI, which peaked at 2 % ((25.5±0.1) min) and remained significantly enhanced at 10 % (Fig.1b). According to our results, moderate pomace addition (~2 %) most effectively stabilises emulsions by forming a cohesive interfacial film with optimal viscosity. Further increases in particle content appear to disrupt the film or hinder protein redistribution.

### Antioxidant properties

The enrichment of SPCs with black mulberry pomace significantly (p<0.001) enhanced their *in vitro* antioxidant properties, as evidenced by increased total flavonoid content (TFC), total phenolic content (TPC), ABTS and DPPH radical scavenging activities, and Fe(II) chelation capacity (Fig. 2). The highest TFC, expressed as QE, was observed at the 2 % pomace inclusion (752.31 µg/g; Fig. 2a), while TPC, expressed as GAE, reached a maximum at 5 % pomace (27.80 mg/g; Fig. 2b). Both ABTS and DPPH activities increased with the enrichment from 1 to 5 %, followed by a slight decrease at 10 % (Fig. 2c and Fig. 2d). In contrast, Fe(II) chelating activity peaked at 1 % and decreased at higher pomace mass fractions (Fig. 2e).

**Fig. 2:**
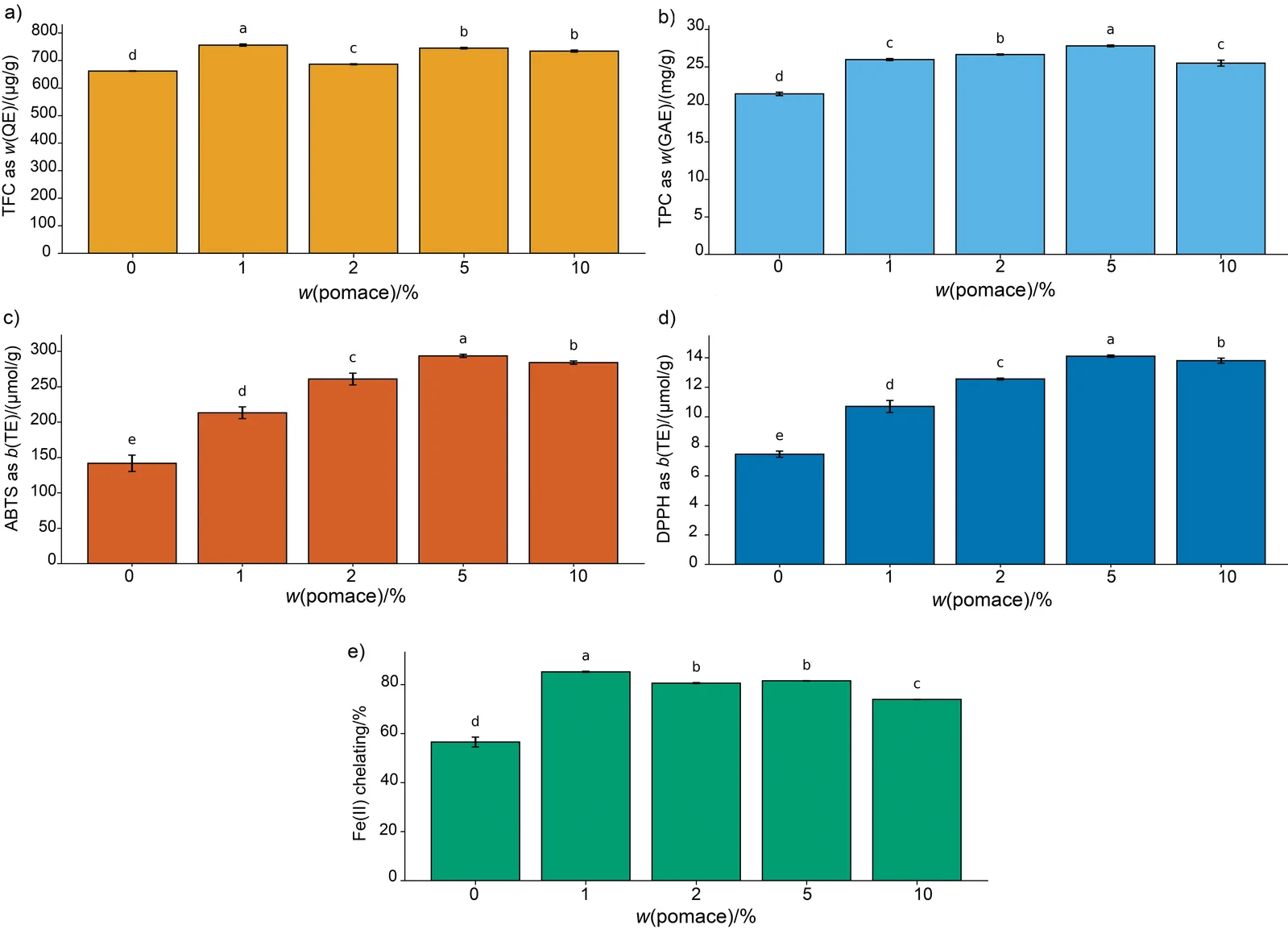
Antioxidant properties of soy protein concentrates with 0–10 % black mulberry pomace: a) total flavonoid content (TFC), b) total phenolic content (TPC), c) ABTS radical scavenging activity, d) DPPH radical scavenging activity, and e) Fe(II) chelating activity. Values are expressed as mean±S.D. Different letters above bars indicate significant differences (p<0.05)

These results emphasise the contribution of phenolic compounds from black mulberry pomace to the antioxidant potential of SPCs. However, the non-linear trends, particularly the decrease at 10 % enrichment, are consistent with two phenomena reported in protein-polyphenol systems: (*i*) a masking effect, in which the binding of phenolics to proteins reduces measurable antioxidant capacity due to the reduced accessibility of phenolic -OH groups to radicals (*36*-*38*), and (*ii*) the formation of aggregated or insoluble complexes at high phenolic loads, which limits their dispersion and reactivity in solution assays (*38*). For example, binding of epigallocatechin gallate or gallic acid to β-casein reduced ABTS radical-scavenging activity by ~20 %, and by ~21 % when bound to albumin (*36*). Also, β-casein-chlorogenic acid complexes exhibited lower ABTS radical-scavenging activity than the sum of their free forms, while FRAP values showed a synergistic effect, indicating assay-dependent differences in antioxidant outcomes (*36*). These findings suggest that moderate pomace enrichment (1–5 %) optimises antioxidant activity, while higher mass fractions may diminish it due to structural constraints and phenolic overloading.

### In vitro digestibility

While antioxidant properties reflect the bioactive potential of SPCs enriched with black mulberry pomace, their nutritional quality also depends on protein accessibility during digestion. Therefore, *in vitro* digestibility was assessed to evaluate the impact of phenolic compounds, dietary fibre, and their interactions with proteins on enzymatic hydrolysis efficiency. As shown in Fig. 3, the *in vitro* protein digestibility of soy protein concentrates was significantly (p<0.05) influenced by the incorporation of black mulberry pomace.

**Fig. 3:**
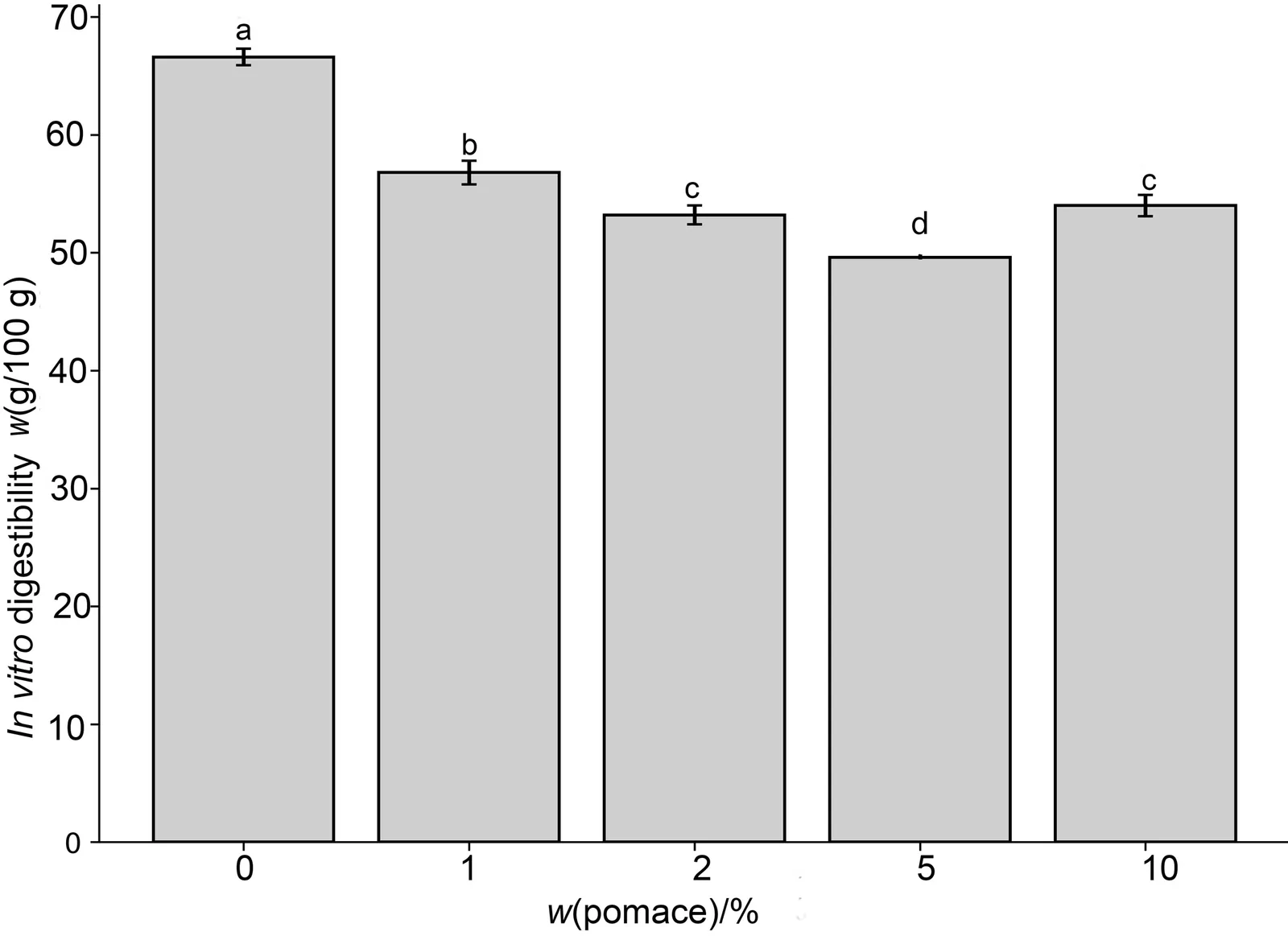
*In vitro* digestibility of soy protein concentrates enriched with black mulberry pomaces. Values are expressed as mean±S.D. Different letters above bars indicate significant differences (p<0.05)

The control sample (0 %) exhibited the highest digestibility. With increasing pomace content, digestibility progressively declined, reaching a minimum at 5 %, followed by a slight increase at 10 %. However, all values remained significantly (p<0.05) lower than the control. The reduction in *in vitro* digestibility with increasing pomace mass fractions is consistent with previous reports (*39*, *40*), which indicate that polyphenols can form stable complexes with proteins, thereby reducing their susceptibility to proteolytic enzymes. Such interactions occur through hydrogen bonding, hydrophobic forces, and, in some cases, covalent linkages. This may lead to protein aggregation or the formation of insoluble matrices that limit enzyme accessibility to peptide bonds. In addition, the increase in water-holding capacity, especially at 1 % enrichment (Fig. 1c), may indicate enhanced hydration yet also suggest a denser protein-fibre network that impedes enzyme diffusion. Furthermore, the accumulation of insoluble carbohydrates with increasing pomace mass fractions may further contribute to the physical encapsulation of proteins, thereby reducing their availability for proteolytic digestion.

Despite the observed reduction in digestibility, the inclusion of mulberry pomace enhances the nutritional profile of SPCs by increasing dietary fibre, phenolic compounds, and antioxidant capacity. Therefore, in designing functional food products, a balance should be sought between protein digestibility and the incorporation of health-promoting bioactive components.

### Colour analysis

In addition to affecting nutritional accessibility, the incorporation of black mulberry pomace also altered the visual characteristics of SPCs. Colour was analysed to quantify these changes and to explore the influence of phenolic pigments, particularly anthocyanins, and their interactions with the protein-fibre matrix on the chromatic attributes of the samples. The progressive incorporation of black mulberry pomace into soy protein concentrates resulted in significant (p<0.05) changes in colour attributes (Table 2). Lightness (L*) values decreased markedly from 90.29 in the control sample to 71.72 in the 10 % formulation, indicating sample darkening. The a* values (red–green axis) shifted from -0.65 (slightly green) to 1.90, reflecting increased redness with higher pomace mass fractions. In contrast, the b* values (yellow–blue axis) remained relatively stable across all treatments.

**Table 2:** Colour parameters of soy protein concentrates enriched with mulberry pomace

*w*(pomace)/%	L*	a*	b*	C*	*h°*	Δ*E*
0	(90.3±0.3)^a^	(-0.6± 0.1)^e^	(11.3±0.4)^b^	(11.3±0.4)^b^	(93.3±1.1)^a^	0.000^d^
1	(82.7±0.4)^b^	(0.51±0.07)^d^	(12.4±0.4)^a^	(12.4±0.4)^a^	(87.6±1.1)^b^	(7.7±0.3)^c^
2	(82.2±0.5)^b^	(0.26±0.09)^c^	(10.7±0.4)^b^	(10.7±0.4)^b^	(88.6±1.1)^b^	(8.2±0.4)^c^
5	(76.0±0.4)^c^	(1.12±0.06)^b^	(10.6±0.4)^b^	10.6±0.4)^b^	(84.0±1.2)^c^	(14.4±0.4)^b^
10	(71.7±0.3)^d^	(1.89±0.07)^a^	(12.2±0.4)^a^	(12.3±0.4)^a^	(81.1±1.2)^c^	(18.8±0.5)^a^

Values are expressed as mean±S.D. Colour parameters include lightness (L*), red-green coordinate (a*), yellow-blue coordinate (b*), chroma (C*), hue angle (*h°*), and total colour difference (Δ*E*)

To further clarify the origin of these colour changes, monomeric anthocyanins were quantified using the pH differential method. They were detected only in the 5 and 10 % pomace-enriched SPCs (0.008 and 0.50 mg/100 g, respectively; data not shown). This indicates that most anthocyanins were present in bound or polymerised forms. Such forms are typically stabilised through non-covalent interactions with proteins and polysaccharides (*via* hydrogen bonding, hydrophobic and electrostatic forces) and are not pH-responsive (*19*, *41*, *42*). Despite their lack of detection by this assay, bound and polymerised anthocyanins can still contribute to visible redness (increase in a*), darkening (decrease in L*), and overall colour change (increase in *ΔE*) through pigment-matrix associations and co-pigmentation with other phenolics. For mulberry anthocyanins, phenolic-acid co-pigmentation has been shown to intensify colour and improve stability. Chen *et al*. (*43*), for example, demonstrated that intermolecular co-pigmentation of mulberry anthocyanins with phenolic acids (*e.g.* ferulic and caffeic acids) significantly enhanced colour intensity (*e.g*. ~53 % hyperchromic effect) and stability *via* mechanisms such as hydrogen bonding and π–π stacking. At the highest pomace mass fraction, the saturation of binding sites may have allowed a small fraction of anthocyanins to remain free, thereby becoming detectable by the pH differential method.

### FTIR analysis

FTIR spectroscopy (Fig. 4) was used to investigate structural modifications in SPCs induced by the incorporation of black mulberry pomace. Across all samples, the FTIR spectra (Fig. 4a and Fig. 4b) displayed well-defined absorption features typical of protein-rich plant matrices, with a broad O-H stretching band near 3300 cm^-1^, C-H stretching vibrations around 2925 cm^−1^, and prominent amide bands at ~1650 cm^-1^ (amide I), ~1540 cm^-1^ (amide II), and ~1240 cm^-1^ (amide III) (*27*, *44*). These signals collectively reflect contributions from the polypeptide backbone of soy proteins, hydroxyl-bearing phenolic constituents, and carbohydrate-derived structures such as pectins and hemicelluloses.

**Fig. 4:**
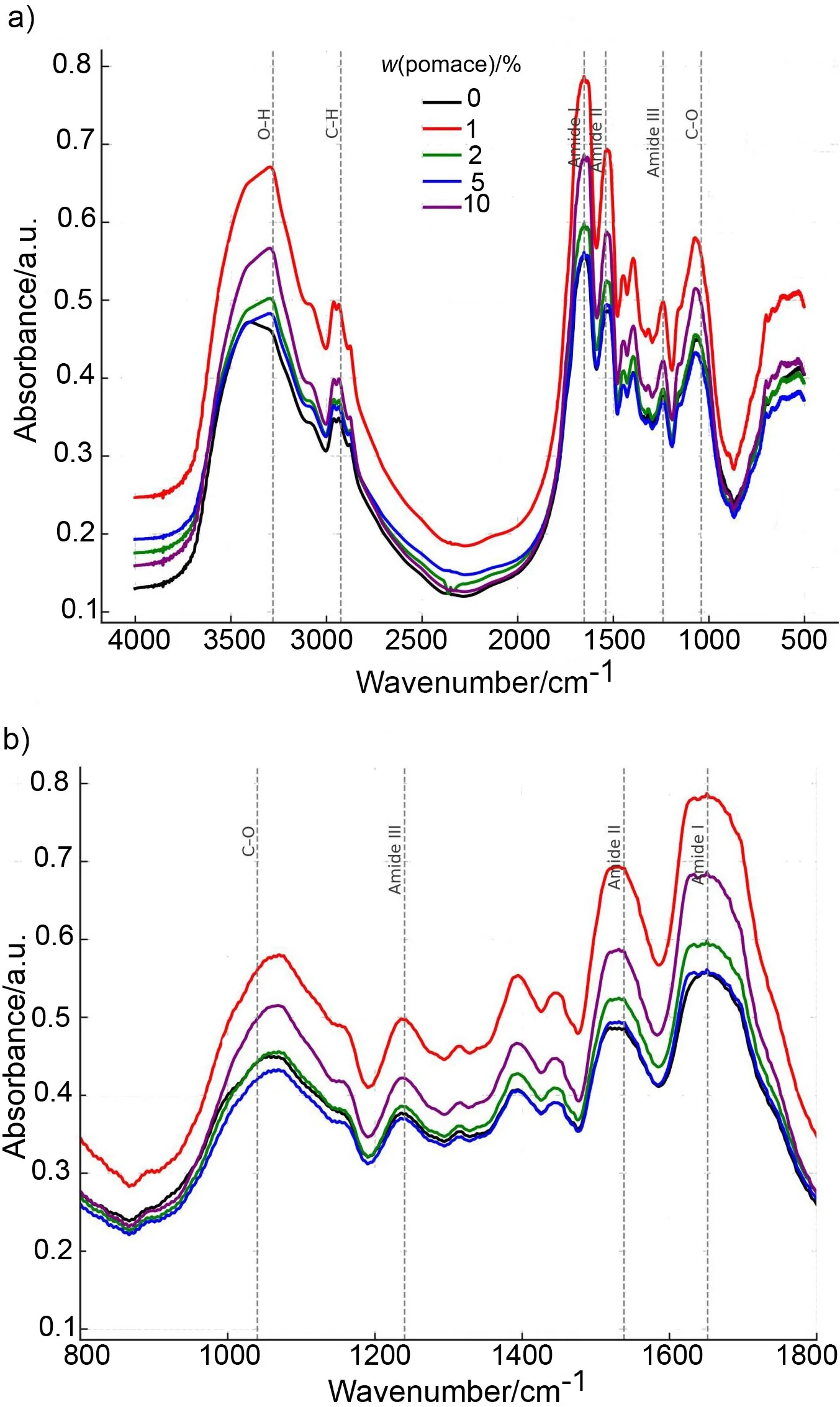
FTIR spectra of soy protein concentrates with 0–10 % black mulberry pomace: a) full spectra (4000–600 cm^−1^), b) expanded region highlighting main absorption bands

Significant shifts were observed (Fig. 4 and Table S1). The O-H band exhibited a pronounced red shift (-104 cm^-1^ at 10 % pomace), indicative of enhanced hydrogen bonding with pomace-derived hydroxyl groups. The amide I band remained unshifted (Δ*ν*=0 cm^-1^), suggesting preservation of the global α-helix/β-sheet composition. The amide II band shifted upwards by approx. 12 cm^-1^, indicating alterations in N-H bending and C-N stretching consistent with modification of the protein hydrogen bonding network due to protein-polyphenol and protein-fibre interactions (*43*). The *I*_1650_/*I*_1540_ ratio remained nearly constant (1.15 *vs* 1.17; Table S1), implying no substantial change in secondary structure, although minor rearrangements cannot be ruled out. Changes in the amide III and C-O bands suggest integration of pomace polysaccharides (*e.g.* pectins, hemicellulose) into the SPC matrix. These findings align with well-established FTIR interpretive principles: amide I (mainly C=O stretching) and amide II (N-H bending and C-N stretching) are sensitive to secondary structure and hydrogen bonding, although amide II is less quantitative than amide I (*44*).

FTIR results indicate that the addition of mulberry pomace strengthens hydrogen-bonded networks and causes minor conformational shifts in SPCs. At 1 % pomace, the red shift of O-H and the upshift of amide II bands correlated with the highest WHC, suggesting that stronger hydrogen bonding increased the number of hydrophilic binding sites. Changes in the amide II and C-O stretching region (~1050–1150 cm^-1^) also indicate the exposure of hydrophobic domains and the incorporation of carbohydrate structures, both relevant for interfacial activity. At low pomace mass fractions (1–2 %), these adjustments appear to promote protein adsorption and film formation at the oil–water interface, thereby supporting a higher emulsifying capacity and stability. At higher mass fractions (5–10 %), stronger hydrogen bonding and matrix rigidity may restrict protein flexibility and diffusion. This can explain the decrease in EAI relative to 1 %, while remaining above the control, despite stable ESI.

### Microstructure (SEM) and relation to FTIR and functional properties

Scanning electron micrographs (SEM) revealed progressive microstructural changes in SPCs after the incorporation of pomace (Fig. 5). The control (Fig. 5a) displayed relatively compact protein domains with smooth surfaces. At 1 % addition, the matrix appeared more open and discretized with fine pores and discontinuities enhancing water access (Fig. 5b). This morphology aligns with the highest WHC observed at 1 % (Fig. 1). Consistently, FTIR spectra (Fig. 4) showed protein backbone bands together with broad O-H/N-H stretching and C-H stretching regions, reflecting contributions of phenolics and carbohydrate moieties.

**Fig. 5:**
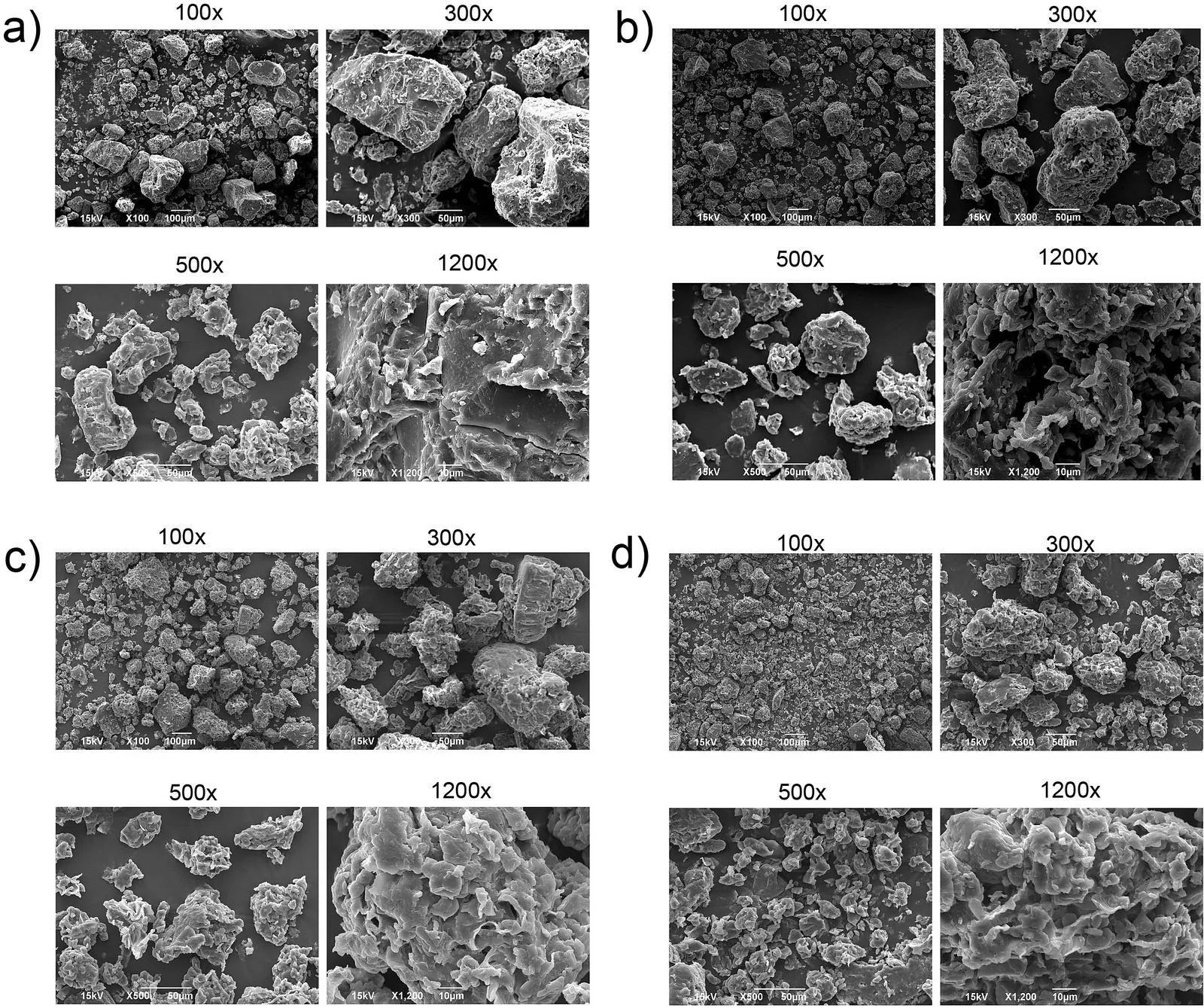
Scanning electron micrographs (SEM) of soy protein concentrates with *w*(black mulberry pomace): a) 0, b) 1, c) 5, and d) 10 %. Each panel shows images at 100×, 300×, 500× and 1200× magnification

SEM images of the samples with 5 % pomace (Fig. 5c) showed thicker lamellae and incipient agglomeration of particulates embedded within the protein matrix. This coincided with partial recovery of WHC and OHC (Fig. 1), suggesting capillary retention within fibre-rich domains and exposure of hydrophobic patches capable of binding oil. FTIR confirmed the incorporation of carbohydrate- and phenol-associated structures. The heterogeneity observed at this inclusion level, with porous corridors juxtaposed against denser clusters, may favour water and oil retention while maintaining interfacial stability.

SEM of the samples with 10 % pomace (Fig. 5d) indicated pronounced aggregation and dense, compacted regions interspersed with larger voids. This morphology corresponds to reduced EAI and a plateau in antioxidant activity (Fig. 2). Increased viscosity and reduced protein mobility likely limited interfacial adsorption. FTIR spectra remained dominated by amide bands, but enhanced O-H and carbohydrate-associated bands suggested tighter polyphenol-polysaccharide-protein associations. These complexes, together with denser domains, likely hindered enzyme penetration, explaining the reduced digestibility (Fig. 3).

Microstructural and chemical associations can also explain colour changes. Increasing pomace reduced L* while increasing a* and chroma values. Anthocyanin-rich particles, visible in SEM as darker inclusions (5–10 %), contributed to both light absorption/scattering and to the broad O-H band. Their co-localisation within compacted regions may stabilise colour but simultaneously reduce enzyme accessibility.

### Correlation and multivariate analysis

To integrate the results of this investigation and gain a comprehensive understanding of the interrelationships between individual parameters, correlation analysis (Fig. S1), principal component analysis (PCA; Fig. 6a and Fig. 6b), and hierarchical cluster analysis (HCA; Fig. 6c) were applied.

**Fig. 6:**
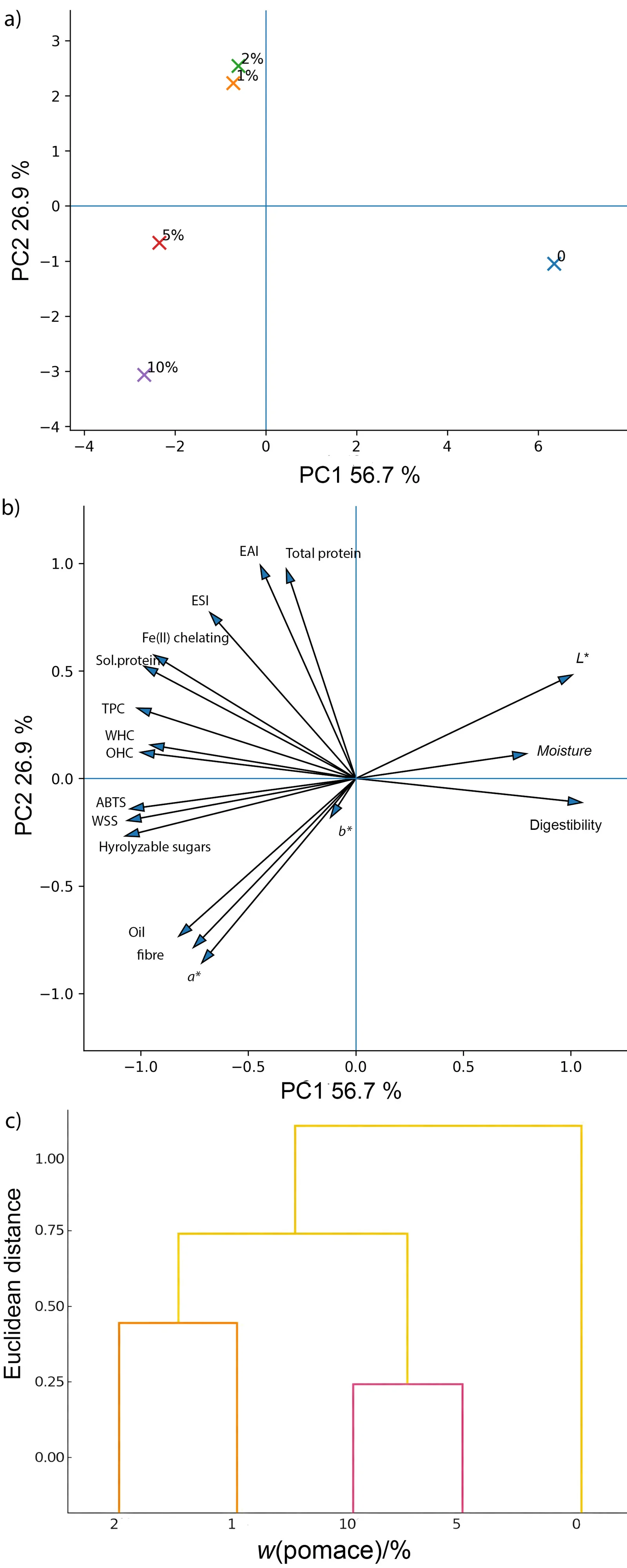
Multivariate analysis of soy protein concentrates with *w*(black mulberry pomace)=0, 1, 2, 5 and 10 %: a) PCA score plot, b) PCA loading plot, and c) hierarchical cluster analysis (HCA) dendrogram. EAI=emulsion activity index, ESI=emulsion stability index, WSS=water-soluble sugar, WHC=water-holding capacity, OHC=oil-holding capacity, TFC=total phenol content, TFC=total flavonoid content, L*=lightness, a*=red-green coordinate, b*=yellow-blue coordinate, ABTS=radical scavenging activity

Pearson’s correlation analysis showed clear relationships between the chemical composition, techno-functional properties, antioxidant activity, and colour parameters of soy protein concentrates enriched with black mulberry pomace. DPPH and ABTS radical-scavenging activities were perfectly correlated (r=1.00). Both parameters showed strong positive correlations with TPC (r=0.88), while correlations with TFC were moderate (r=0.59-0.61). Water-soluble and acid-hydrolysable sugars showed very strong positive correlations with DPPH and ABTS (r=0.97-1.00), suggesting that sugar-rich matrices co-varied with enhanced antioxidant responses. In contrast, *in vitro* protein digestibility showed very strong negative correlations with DPPH and ABTS (r=-0.96 for both), TPC (r=-0.97), and WS/HS sugars (r=-0.94 and -0.91, respectively), indicating that polyphenol-protein and fibre-protein interactions may limit enzymatic accessibility. Among functional properties, total protein content correlated very strongly with ESI (r=0.92) and strongly with EAI (r=0.81), whereas its relationship with WHC was weak (r=0.29).

According to the results of this study, soluble protein emerged as a key parameter that showed very strong positive correlation with Fe(II) chelating capacity (r=0.99) and strong correlations with WHC and OHC (both r=0.84). Strong correlations with DPPH and ABTS activities (r=0.76 for both) were also observed. In addition, soluble proteins were very strongly and negatively correlated with digestibility (r=-0.87), highlighting their role in both antioxidant potential and protein accessibility.

Colour parameters closely depended on the composition of SPCs. Fibre and oil contents were very strongly correlated with redness (a*; r=0.98/0.97) and very strongly and negatively correlated with lightness (L*; r=-0.89/-0.98). Digestibility showed a moderate positive association with L* (r=0.65) and a weaker negative correlation with a* (r=-0.49). These co-variations consistently indicate that mulberry pomace enrichment reshapes the protein-polyphenol-fibre network, simultaneously influencing antioxidant responses, colour and digestibility.

Principal component analysis (PCA; Fig. 6a and Fig. 6b) was applied to reduce the dimensionality of the dataset and elucidate the relationships among physicochemical, functional, antioxidant and colour parameters of soy protein concentrates enriched with black mulberry pomace. The first two principal components explained 83.6 % of the total variance, with PC1 accounting for 56.7 % and PC2 for 26.9 %, indicating that these components adequately describe the variability within the dataset.

PC1 primarily represented a compositional and biofunctional gradient that clearly separated samples according to pomace mass fraction. Samples enriched with higher mass fractions of pomace were positioned on the negative side of PC1 and were associated with increased total phenolic content, antioxidant activity (ABTS, DPPH and TAC), oil and fibre contents, as well as higher oil-holding capacity. In contrast, the control sample was located on the positive side of PC1, where it was associated with higher *in vitro* digestibility, moisture content, and higher L* values, indicating a lighter colour.

PC2 further contributed to the differentiation of samples and was influenced by a combination of protein-related, functional, and colour parameters. The positive side of PC2 was associated with higher protein-related functionality and emulsifying properties, while negative PC2 values were linked to compositional characteristics such as fibre and lipid fractions, as well as colour intensity. Together, these results indicate that sample distribution in the PCA space was governed by a combination of compositional, functional, and colour-related factors, with a clear distinction between protein functionality and fibre-rich fractions.

Hierarchical cluster analysis (HCA; Fig. 6c) supported the PCA results and confirmed the grouping pattern of the samples. The samples with 1 and 2 % pomace formed one subcluster, indicating a high degree of similarity in their overall compositional and functional profiles, while the samples with 5 and 10 % pomace formed a second, even more closely related subcluster. These two pomace-containing subclusters were further grouped together, while the control sample remained clearly separated, confirming its distinct physicochemical and functional profile.

PCA and HCA provide an integrated statistical perspective, corroborating trends observed in individual parameters, FTIR and SEM analyses, and correlation patterns. The clustering of samples along biofunctional and compositional axes reinforces the conclusion that even low pomace mass fractions substantially alter the nutritional and functional properties of SPCs.

## CONCLUSIONS

This study demonstrated that the incorporation of black mulberry pomace into soy protein concentrates induces pronounced compositional, structural, and functional modifications. Moderate enrichment, particularly at lower mass fractions, enhanced hydration and emulsifying properties, while simultaneously improving antioxidant capacity. These findings highlight the potential of mulberry pomace as a sustainable ingredient for tailoring the techno-functional profile and bioactivity of soy protein concentrates. The novelty of this work lies in linking microstructural and spectroscopic evidence with functional performance, thereby offering new perspectives on the valorisation of fruit by-products in plant-based protein formulations. Future studies should focus on optimising the mass fraction of added pomace in relation to protein digestibility and on evaluating the performance of these concentrates in real food systems, in order to further support their application as sustainable functional ingredients.

## Figures and Tables

**Fig. S1:**
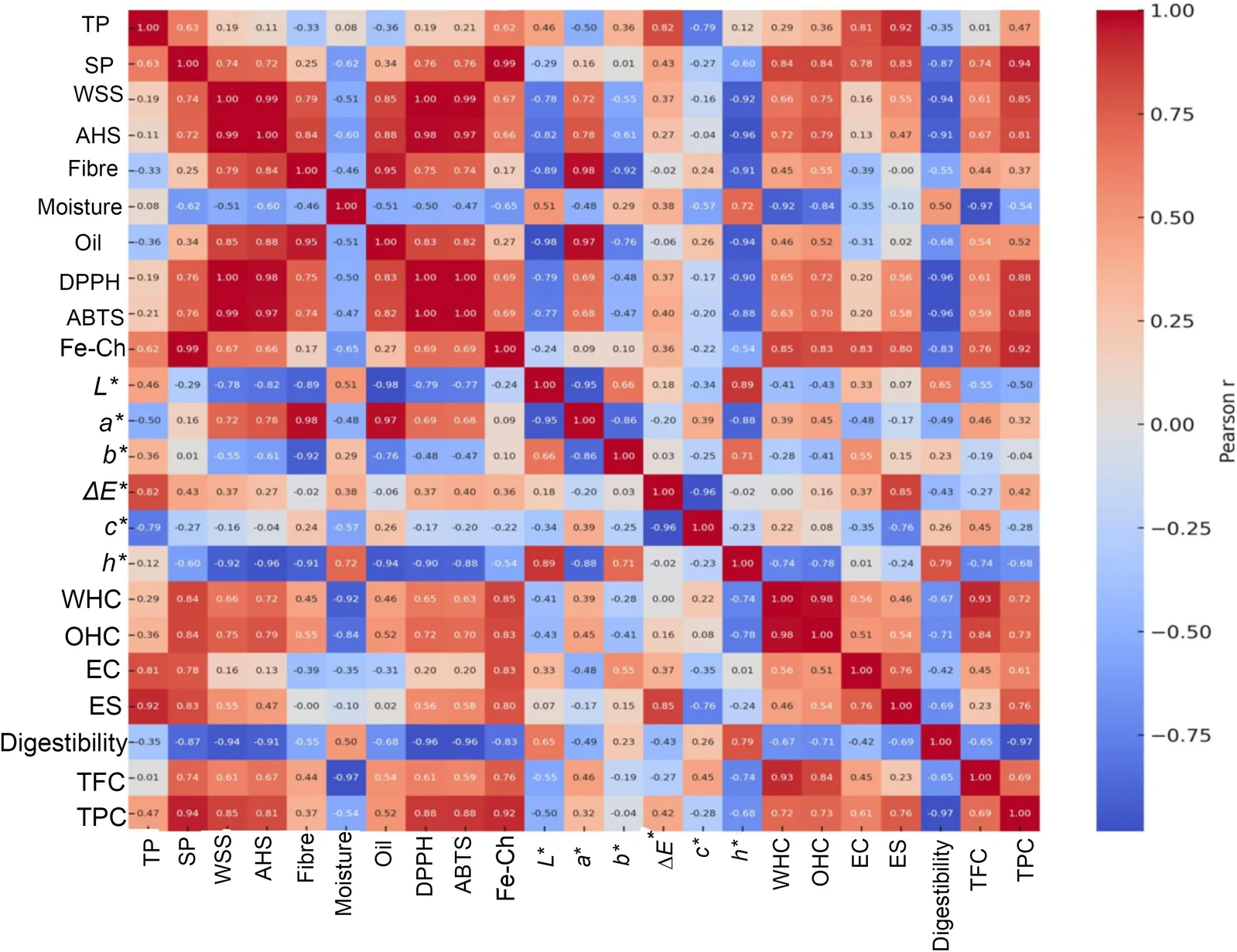
Correlation heatmap showing Pearson correlation coefficients (p<0.05) between chemical composition, techno-functional, antioxidant and digestibility parameters of soy protein concentrates with 0–10 % black mulberry pomace. TP=total protein, SP=soluble protein, WSS=water-soluble sugar, AHS=acid-soluble sugar, ABTS=radical scavenging activity, Fe-Ch=Fe(II) chelating ability, WHC=water-holding capacity, OHC=oil-holding capacity, DPPH=radical scavenging activity, TFC=total phenol content, TFC=total flavonoid content, L*=lightness, a*=red-green coordinate, b*=yellow-blue coordinate, C*=chroma, *h°=*hue angle, Δ*E=*total colour difference

## Appendix

**Table S1:** Characteristic FTIR absorption bands of soy protein concentrates with different mass fractions of mulberry pomace and their shifts relative to the control (0 % pomace)

Functional group (Region/cm^−1^)	*ν*_max_(P_0_)/cm^−1^	Δ*ν*(P_0_ *vs* P_0_)/cm^−1^	*ν*_max_(P_1_)/ cm^−1^	Δ*ν*(P_1_ *vs* P_0_)/ cm^−1^	*ν*_max_(P_2_)/ cm^−1^	Δ*ν*(P_2_ *vs* P_0_)/cm^−1^	*ν*_max_(P_5_)/cm^−1^	Δ*ν*(P_5_ *vs* P_0_)/cm^−1^	*ν*_max_(P_10_)/cm^−1^	Δ*ν*(P_10_ *vs* P_0_)/cm^−1^
O–H (3700–3000)	3400.50	0.00	3292.49	-108.01	3294.42	-106.08	3294.42	-106.08	3296.35	-104.16
C–H (2970–2850)	2933.73	0.00	2960.73	27.00	2960.73	27.00	2958.80	25.07	2933.73	0.00
Amide I (1700–1600)	1651.07	0.00	1651.07	0.00	1651.07	0.00	1651.07	0.00	1651.07	0.00
Amide II (1580–1480)	1519.91	0.00	1531.48	11.57	1531.48	11.57	1531.48	11.57	1531.48	11.57
Amide III (1300–1200)	1236.37	0.00	1236.37	0.00	1236.37	0.00	1234.44	-1.93	1236.37	0.00
C–O (1150–1000)	1055.06	0.00	1070.49	15.43	1068.56	13.50	1068.56	13.50	1068.56	13.50
*I*_1650_/*I*_1540_	1.15		1.13		1.14		1.14		1.17	

Negative values indicate a shift to lower wavenumbers than the control (0 % pomace). P_0_, P_1_, P_2_, P_5_ and P_10_=mass fractions of added pomace (0, 1, 2, 5 and 10 %)
